# Hypercholesterolemia-induced increase in plasma oxidized LDL abrogated pro angiogenic response in kidney grafts

**DOI:** 10.1186/s12967-018-1764-4

**Published:** 2019-01-14

**Authors:** Thomas Kerforne, Frédéric Favreau, Tackwa Khalifeh, Souleymane Maiga, Geraldine Allain, Antoine Thierry, Manuel Dierick, Edouard Baulier, Clara Steichen, Thierry Hauet

**Affiliations:** 1 INSERM U1082 IRTOMIT, 2 rue de la Milétrie, CS90577, 86000 Poitiers, France; 2 Service d’Anesthésie-Réanimation, CHU de Poitiers, 86000 Poitiers, France; 3 Faculté de Médecine et de Pharmacie, Université de Poitiers, 86000 Poitiers, France; 4 Faculté de Médecine, EA 6309 “Maintenance Myélinique et Neuropathies Périphériques», Université de Limoges, 87000 Limoges, France; 5 Laboratoire de Biochimie et Génétique Moléculaire, CHU de Limoges, 87000 Limoges, France; 6 Service Medico-Chirurgical de Pediatrie, CHU de Poitiers, 86000 Poitiers, France; 7 Service de Chirurgie Cardio-Thoracique, CHU de Poitiers, 86000 Poitiers, France; 8 Service de Néphrologie et Transplantation, CHU de Poitiers, 86000 Poitiers, France; 9 UGCT-Department of Physics and Astronomy, Faculty of Sciences, Ghent University, Proeftuinstraat 86, 9000 Ghent, Belgium; 10 Service de Biochimie, CHU de Poitiers, Poitiers, 86000 France; 11 IBiSA ‘Plate-Forme MOdélisation Préclinique-Innovations Chirurgicale et Technologique (MOPICT)’, Domaine Expérimental du Magneraud, 17700 Surgères, France; 12 FHU SUPORT ‘SUrvival oPtimization in ORgan Transplantation’, 86000 Poitiers, France

**Keywords:** Oxidized LDL, Kidney transplantation, Vascular remodeling

## Abstract

**Background:**

Renal transplantation is increasingly associated with the presence of comorbidity factors such as dyslipidemia which could influence the graft outcome. We hypothesized that hypercholesterolemia could affect vascular repair processes and promote post-transplant renal vascular remodeling through the over-expression of the anti-angiogenic thrombospondin-1 interacting with vascular endothelial growth factor-A levels.

**Methods:**

We tested this hypothesis in vitro, in vivo and in a human cohort using (1) endothelial cells; (2) kidney auto-transplanted pig subjected (n = 5) or not (n = 6) to a diet enriched in cholesterol and (3) a renal transplanted patient cohort (16 patients).

**Results:**

Cells exposed to oxidized LDL showed reduced proliferation and an increased expression of thrombospondin-1. In pigs, 3 months after transplantation of kidney grafts, we observed a deregulation of the hypoxia inducible factor 1a—vascular endothelial growth factor-A axis induced in cholesterol-enriched diet animals concomitant with an overexpression of thrombospondin-1 and a decrease in cortical microvessel density promoting vascular remodeling. In patients, hypercholesterolemia was associated with decreased vascular endothelial growth factor-A plasma levels during early follow up after renal transplantation and increased chronic graft dysfunction.

**Conclusions:**

These results support a potential mechanism through which a high fat-diet impedes vascular repair in kidney graft and suggest the value of controlling cholesterolemia in recipient even at the early stage of renal transplantation.

**Electronic supplementary material:**

The online version of this article (10.1186/s12967-018-1764-4) contains supplementary material, which is available to authorized users.

## Background

In renal transplantation, the increase of older recipients due to population ageing amplifies the current organ shortage, promoting a raise in the mean donor age. These increases induce a higher prevalence of comorbidity factors which could influence renal graft outcome. During the transplantation process, kidney grafts are unavoidably exposed to ischemia–reperfusion (I/R) injury. The key role played by I/R in defining the balance between regenerative or detrimental pathways and irreversibly programming graft outcome imply that it is of paramount importance to determine the factors or co-morbidity factors which could early interact with graft viability.

Risk factors for cardiovascular diseases such as diabetes, hypertension and elevated plasmatic low-density lipoprotein (LDL) levels are well documented in renal diseases progression and also in transplantation or renal artery stenosis injury [1]. The high prevalence of dyslipidemia combined with the use of older donors and with the hyperlipidemic effects of immunosuppressors strengthens the link between hypercholesterolemia and kidney transplantation [2, 3]. Thus, hyperlipidemia in the recipient is of greater importance related to calcineurin or mTOR inhibitor exposure given the subsequent exposure length of the graft to the recipient milieu. In general, hypercholesterolemia is associated with increased systemic levels of oxidized low density lipoproteins (OxLDL) well known to be involved in endothelial cell dysfunction [4, 5]. Renal microcirculation, one of the critical targets of I/R injury, plays a crucial role in the early regeneration phase but also in the extension of chronic renal disease through a complex interplay between proliferation, regeneration and capillary loss [6–8]. In healthy organs, vascular endothelial cells are protected from moderate injury by the release of autocrine signaling molecules such as vascular endothelial growth factor-A (VEGF-A) [9]. However, endothelial damage induced by I/R affect this regenerative response [10, 11].

Previously, Cui et al. reported the role of thrombospondin-1 (TSP-1) as an important mediator for high fat-diet induced kidney dysfunction in mice characterized by macrophage infiltration and fibrosis development [12]. TSP-1 is also involved in numerous detrimental processes induced by kidney I/R, but little is known about this effect in kidney graft outcome and the link between TSP-1 and OxLDL [13]. TSP-1 is a matricellular glycoprotein that interacts with different receptors and has multiple functions including promotion of clot formation or fibrosis and is also well-known as an inhibitor of angiogenesis by different mechanisms such as interacting with VEGF-A levels or modulating the NO availability which may prevent kidney repair [14].

This study is aimed at determining the role of hypercholesterolemia and particularly oxidized LDL (OxLDL) in early vascular regenerative processes occurring after I/R injury in renal transplantation that are distinct from the chronic injury of atherosclerosis induced by dyslipidemia. We hypothesized that OxLDL could impede protective angiogenesis mechanism induced by I/R through the stimulation of TSP-1 expression and its effects on VEGF-A production.

## Methods

### In vitro incubation of OxLDL on human aortic endothelial cells

OxLDL’s effects were evaluated in Human Aortic Endothelial Cells (HAEC) treated with culture medium supplemented or not with OxLDL (25 μg/mL) for 24 h. Briefly, HAEC obtained from Gibco (Saint Aubin, France) were cultured in Medium 200 (M200, Gibco) supplemented with 10% fetal bovine serum (Invitrogen, Saint Aubin, France) in a humidified atmosphere at 5% CO_2_ and 37 °C. Cells were serum starved for 12 h and then treated. After treatment, condition media was removed and cells were collected to western blotting analysis for protein expression of TSP-1 (1/500, Santa Cruz Biotechnology, Santa Cruz, California, USA), ADAMTS-1 (1/500, Santa Cruz Biotechnology, Santa Cruz, California, USA), PhosphoVEGF-R2 (1/1000, Cell signaling, Leiden, Netherlands), and VEGF-R2 (1/1000, Cell signaling, Leiden, Netherlands). TSP-1 levels in the supernatant were quantified by ELISA kit (RnD Systems, Minneapolis, USA). Flow cytometry analysis was used for alpha5beta3 integrin expression on HAEC cells (1/100, Millipore, Billerica, Massachusetts, USA). The effect of recombinant human TSP-1 (0.5 µg/ml; RD System, Lille, France) on alpha5beta3 integrin protein expressions was investigated. Cell proliferation was assessed by the BrDU assay (Cell Proliferation ELISA, BrdU, Roche, USA) following the manufacturer’s guidelines in cells treated with culture medium supplemented or not with OxLDL for 24 h in presence of recombinant VEGF (Gibco) and SiRNa targeted TSP-1 (Qiagen, Courtaboeuf, France).

### Animal model and surgical procedures

Male Large White pigs (Sus Scrofa) were fed with a standard or a high-fat diet (standard diet + 20% lard and 2% cholesterol) immediately after weaning and maintained until euthanasia, i.e. 3 months after transplantation as previously described in previous studies [1, 15]. Renal auto-transplantation model was performed 2 months after weaning as previously described in accordance with the institutional committee for the use and care of laboratory animals (CEEA Poitou–Charentes CE2012-4) [16]. Briefly, the left kidney was removed, flushed with 300 ml of UW preservation solution and preserved at 4 °C in the same solution in static conditions for 24 h. On the day of transplantation, the right kidney was removed and the left kidney grafted mimicking the nephron mass in transplanted situation. Two experimental groups were studied: Normal diet: transplanted kidneys collected 3 months after surgery from animals fed a standard diet (n = 6), High-fat diet: transplanted kidneys collected 3 months after surgery from animals fed a high-fat diet (n = 5). One transplanted high-fat diet pig died before completion of the study due to surgical complications and was not included in data analysis. Urinary and plasma creatinine or proteinuria were measured using an automatic analyzer (Modular, Roche Diagnostic, Meylan, France). Creatinine clearance was calculated by the formula: (Urinary volume × Urinary creatinine level)/plasma creatinine level. Peripheral blood was collected before kidney transplantation, at 3, 7 days and 1, 3 months after reperfusion. Urines were collected at 3, 7 days and 1, 3 months after reperfusion.

### Western blotting, immunohistochemistry and standard light microscopy

These studies were performed on renal tissue from graft kidneys 3 months after transplantation to assess the mechanisms responsible for formation and maintenance of the renal microvasculature and vascular remodeling. We investigated by standard western blotting protocols [17] pro- angiogenic pathways with specific antibodies against hypoxia inducible factor 1a (HIF1a, 1/500, BD Biosciences, San Jose, California, USA), VEGF (1/500, Santa Cruz Biotechnology), Stromal cell-Derived Factor-1 (SDF-1, 1/1000, Abbiotech, San Diego, California, USA), factors involved in endothelial cells proliferation and migration: matrix metalloproteinase 9 (MMP-9, 1/1000, Millipore), alpha5beta3 integrin (1/1000, Millipore), and anti-angiogenic factor: A Disintegrin And Metalloproteinase with Thrombospondin Motif-1 (ADAMTS-1, 1/200, Santa Cruz Biotechnology). Loading controls were β actin (1/20,000, Sigma, St Louis, Missouri, USA). Protein bands were revealed and intensities were quantified using AlphaEase FC software (Alpha Innotech Corporation, San Leandro, CA). Staining evaluations using semiquantitative analyses on cortex samples was performed as previously described and examined under blind conditions by a pathologist and a nephrologist [18]. Briefly, paraffin sections were used for tubulo-interstitial fibrosis evaluation by Masson trichrome staining. Alpha-smooth muscle actin (αSMA, 1/100, Dako, Stockholm, Sweden) expression was assessed by diamino benzidine staining. The microvascular media-to-lumen ratio was measured in αSMA-positive macrovessels under 500 µm in diameter. Tubular atrophy was assessed by Hematoxylin–Eosin–Safran (HES) staining with previously published scoring [19]. Frozen sections were used for TSP-1 (1/100, Santa Cruz Biotechnology), VEGF (1/100, Santa Cruz Biotechnology), HIF1a (1/100, BD Biosciences) expressions in cortical section by immunofluorescence as well as ED-1 antigen marker of macrophages and monocytes (1/100, AbD Serotec, Oxford, UK).

### Apoptotic signals

Apoptotic renal cells were characterized by the TUNEL method using the DeadEnd Fluorometric TUNEL system (Promega, Fitchburg, Massachusetts, USA) as previously shown [20].

### Real-time quantitative PCR

We used RNA extraction kit (Qiagen, Courtaboeuf, France). Genomic DNA was removed using DNA-free kit (Applied Biosystems, Foster City, California, USA) and first-strand reverse transcription (Applied) was performed. Real-Time PCR assays were performed on an ABI Prism 7300 (Applied) with porcine primers adapted for VEGF and TSP-1 mRNA expressions (Additional file 1: Table S1).

### Tissue preparation, acquisition and analysis of images by high resolution micro-computed tomography

Three months post-transplant at the time of sacrifice, the kidney graft was removed and perfused with a saline solution containing 5000 IU/L of heparin. Then, the saline solution was replaced by an intravascular radio-opaque silicone polymer (Microfil MV122; Flow Tech, Carver, Massachusetts, USA). The kidney was immersed in formalin solution at 4 °C and a cylinder biopsy (diameter: 1 cm; depth: 1.5 cm) of the polymer-filled kidney was performed and encased in paraffin. High resolution micro-computed tomography scans were performed, followed by image reconstructions using specific algorithm [21]. The average diameter and spatial density of cortical vascular segments of microvessels were calculated as previously described [17, 22] and the spatial density classified according to diameter as small (< 40 µm), medium (40–80 µm), large (80–120 µm) or very large (> 120 µm) vessels.

### Patients

A prospective cohort study was conducted on 16 kidney transplant patients at the Transplant Unit of the Poitiers University Hospital between January 2010 and June 2010. Adult recipients of a first or second kidney transplant were eligible for enrollment and patients with preemptive graft, infectious complications and early acute graft rejection have been excluded. The blood samples were taken during the normal follow-up of the patient, anonymized and since the study did not require additional blood sampling, an approval from an ethics committee was not required under French law according to the article L.1121-1 of the public health code. Written informed consents were obtained from each patient according to the Declaration of Helsinki. We classified patients in 2 groups related to the plasma cholesterol levels using a threshold of 1.80 g/L. Renal function was assessed by urinary protein excretion and eGFR according to the simplified modification of diet in renal disease formula MDRD. Peripheral blood was collected by venipuncture at day 0 (D0) before kidney transplantation, 1, 3, 7 and 14 days (respectively D1, D3, D7 and D14), 1, 3 and 12 months after surgery. Plasma samples were stored at − 80 °C prior to protein quantification by ELISA for OxLDL (Mercodia, France), and VEGF-A (RD System).

### Statistics

Results are shown as means ± SD. We used a student t-test for two-group comparisons or a Mann–Whitney test when the variance was not equal between the groups. For multiple group comparison, we used Kruskal–Wallis test. Fisher’s exact test was used for statistical analysis of proportions. Statistical significance was accepted for p < 0.05.

## Results

### The inhibition of endothelial cells proliferation induced by OxLDL is mediated by TSP-1

**Fig. 1:**
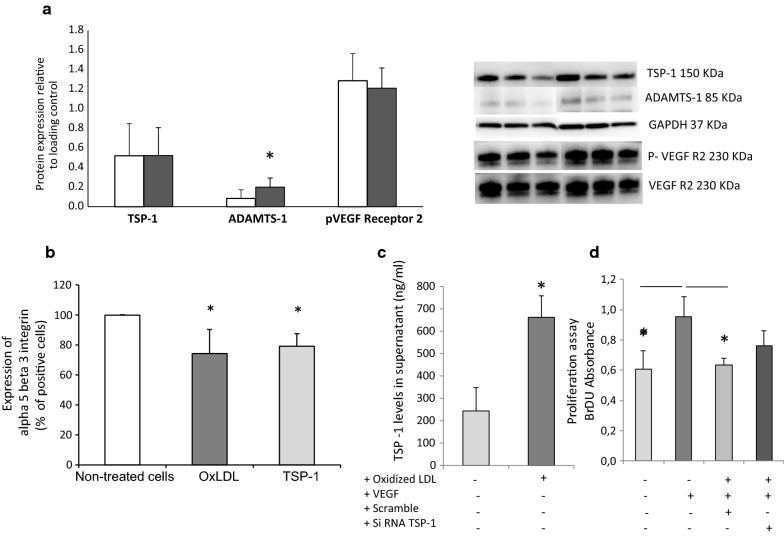
The inhibition of endothelial cells proliferation induced by OxLDL is mediated by TSP-1. OxLDL incubation induced an increase of ADAMTS-1 expression in HAEC without effect on TSP-1 and PhosphoVEGF-R2 protein expression (**a**; N = 3). Alpha5beta3 integrin expression on HAEC was reduced by OxLDL and human recombinant TSP-1 (**b**; N = 3). OxLDL enriched culture medium induced an increases secretion of TSP-1 by HAEC (**c**, N = 3). The inhibition of VEGF-proliferation cells induced by OxLDL was limited by SiRNA targeted TSP-1 (D, N = 4)

OxLDL-enriched culture medium induced the expression of ADAMTS-1 in HAEC and promoted the increase of TSP-1 secretion in supernatant without effect on phosphorylation of VEGF-R2 (Fig. 1a, c). Alpha5beta3 integrin expression on HAEC, marker of endothelial cell proliferation, was decreased in both conditions: in culture medium enriched with OxLDL or supplemented with TSP-1 human recombinant protein (Fig. 1b). Interestingly, SiRNA targeted TSP-1 (Additional file 2: Figure S1) limited the inhibition of cell proliferation induced by OxLDL in presence of VEGF underlining the role of TSP-1 in these inhibited conditions (Fig. 1d).

**Table 1:** Summary table of kidney function and blood metabolites of transplanted animals fed either a normal or a high-fat diet maintained for 3 months after surgery (M3)

	D0	D3	D7	M1	M3
Creatinine clearance (ml/min)					
Normal diet	/	7.8 ± 7.5	22.1 ± 3.3	71.9 ± 22.3	64.5 ± 35.3
High-fat diet	/	8.6 ± 14.3	24.7 ± 26.7	61.4 ± 22.3	58.5 ± 26.1
Urinary ratio protein/creatinine (mg/mmol)					
Normal diet	/	/	/	124 ± 5	108 ± 4
High-fat diet	/	/	/	236 ± 12*	183 ± 9*

Values are mean ± SD

* p < 0.05 vs. normal diet, n = 5–6 in each group

### High-fat diet reduces pro-angiogenic pathways in renal auto-transplanted pig

**Fig. 2:**
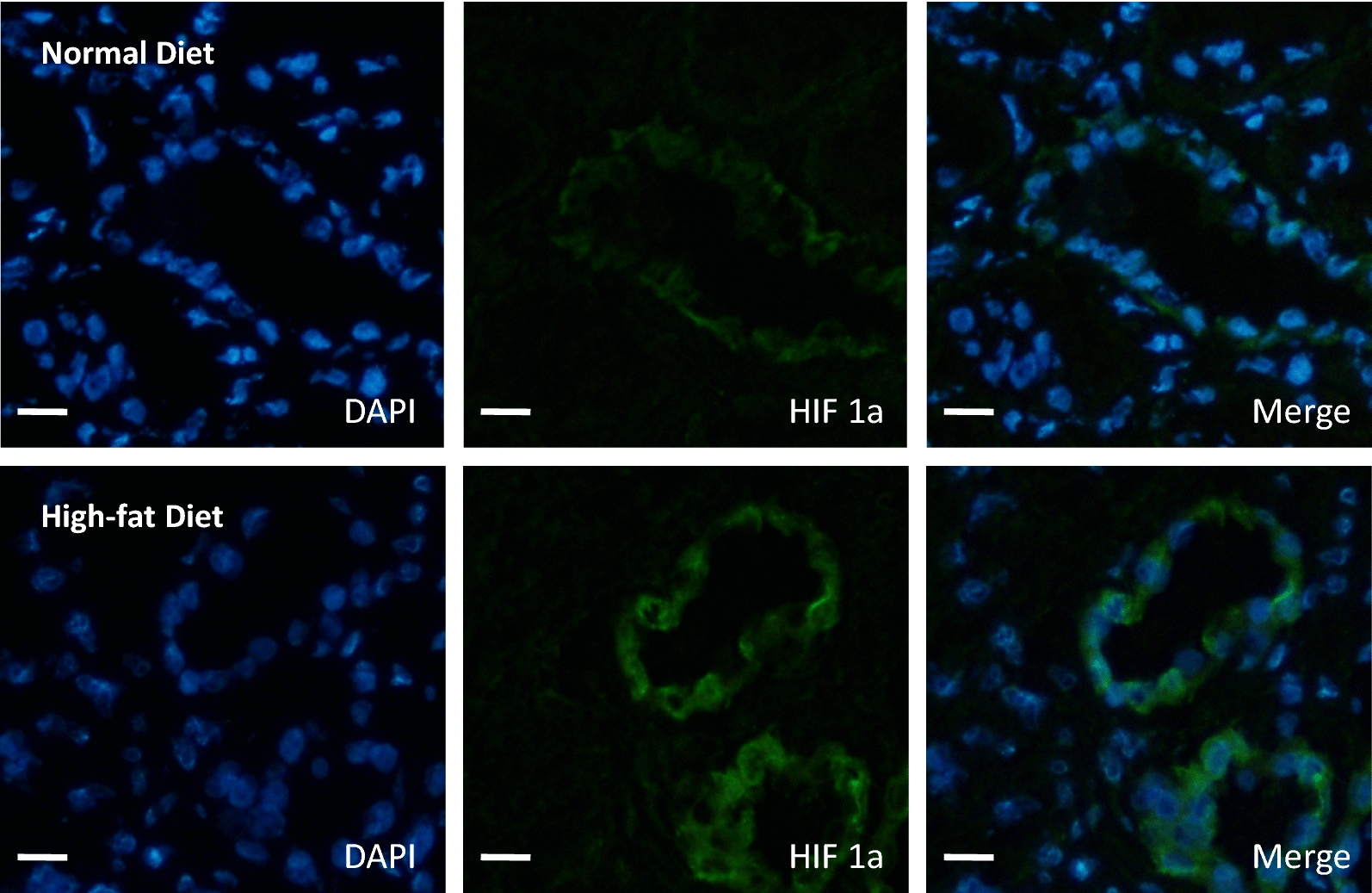
High-fat diet increases HIF1a expression in porcine auto-transplanted kidneys. Immunofluorescence staining in cortex tissue for HIF1a in high-fat or normal diet groups, 3 months after renal auto-transplantation surgery. Scale bars represent 100 µm (magnification ×40; n = 5 in each group)

**Fig. 3:**
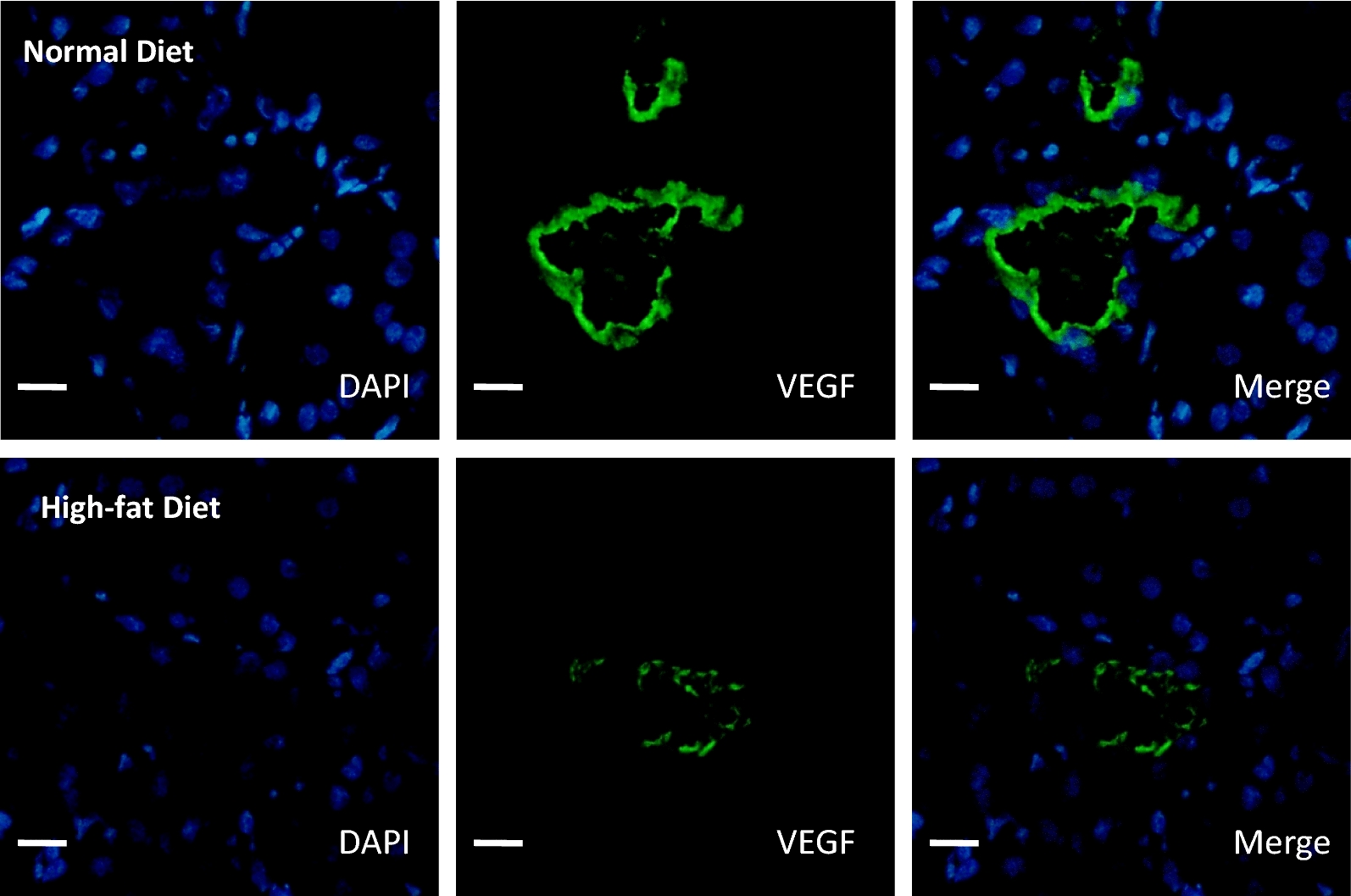
High-fat diet reduces VEGF expression in porcine auto-transplanted kidneys. Immunofluorescence staining in cortex tissue for VEGF in high-fat or normal diet groups, 3 months after renal auto-transplantation surgery. Scale bars represent 100 µm (magnification ×40; n = 5 in each group)

**Fig. 4:**
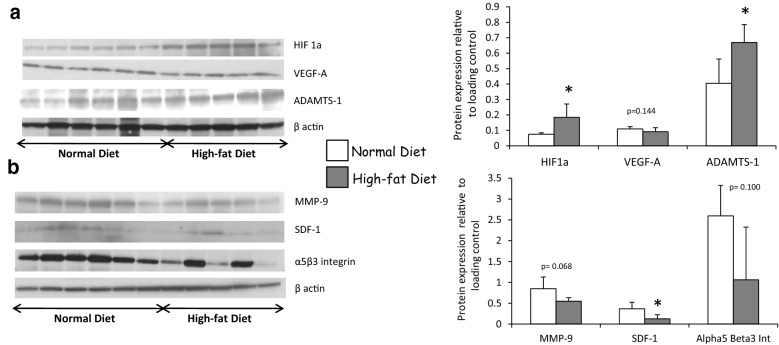
High-fat diet reduces pro-angiogenic pathways in porcine auto-transplanted kidneys. Expression by western blotting of proteins involved in pro-angiogenic pathways: HIF1a, VEGF-A, ADAMTS-1, MMP-9, SDF-1, alpha5beta3 integrin (**a**, **b**). Values significantly different from the normal diet group are represented by *p < 0.05; n = 5 in high-fat diet and n = 6 in normal diet group

In a clinical relevant porcine model, as previously described, hyperlipidic diet induced an increase of plasma cholesterol level in the last part of the follow up even if OxLDL plasmatic levels were significantly elevated at the day of surgical procedure and at 3 months post-transplantation [15]. As previously reported, only high-fat diet was associated with an increased proteinuria at 1 and 3 months while both experimental conditions, with or without high-fat diet, induced similar kinetics for creatinine clearance (Table 1) [15]. The effects of OxLDL on regenerative vascular processes suggested by our in vitro results were further assessed by measuring the expressions of proteins involved in these pathways at 3 months post-reperfusion. The hypoxic pathway mediated by HIF1a well known to promote VEGF production was abrogated by the high-fat diet. Dyslipidemic pigs exhibited an increased expression of HIF1a in kidney grafts 3 months after while VEGF-A expression remained stable or even decreased when assessed by western blotting and immunohistochemistry respectively (Figs. 2, 3, 4a, b, Table 2, Additional file 3: Figure S2, Additional file 4: Figure S3). In addition, high-fat diet increased the expression of ADAMTS-1 (Fig. 4a) known as an activator of the TSP-1 pathway and decreased the expression of proteins involved in endothelial cell proliferation and migration such as SDF-1 (Fig. 4b). A trend was observed for MMP-9 and alpha5beta3 integrin (Fig. 4b).

**Table 2:** Summary table of semi-quantification of kidney stainings in transplanted animals fed either a normal or a high-fat diet maintained for 3 months after surgery (M3)

	Normal diet	High-fat diet
Renal HIF1a expression by field		
Positive tubules	5.40 ± 0.53	11.52 ± 0.58*
Positive cells	5.00 ± 0.63	11.88 ± 1.00*
Surface area (%)	6.00 ± 0.35	13.70 ± 0.91*
Renal VEGF-A expression by field positive vessels	6.98 ± 0.47	2.78 ± 0.32*
Renal TSP-1 expression by field		
Positive glomerulus parietal cell	2.54 ± 0.28	2.72 ± 0.35
Positive glomerulus	1.73 ± 0.29	3.12 ± 0.28*
Positive tubules	2.69 ± 0.47	9.03 ± 1.39*
Interstitium staining (%)	4.00 ± 0.63	16.83 ± 0.70*
Tubular atrophy (%)	6.35 ± 1.30	16.80 ± 0.74*
Trichrome staining (%)	6.31 ± 0.78	16.81 ± 0.47*

Values are mean ± SD

* p < 0.05 vs. normal diet, n = 5 in each group

### TSP-1 pathway is activated by high-fat diet in kidney graft

**Fig. 5:**
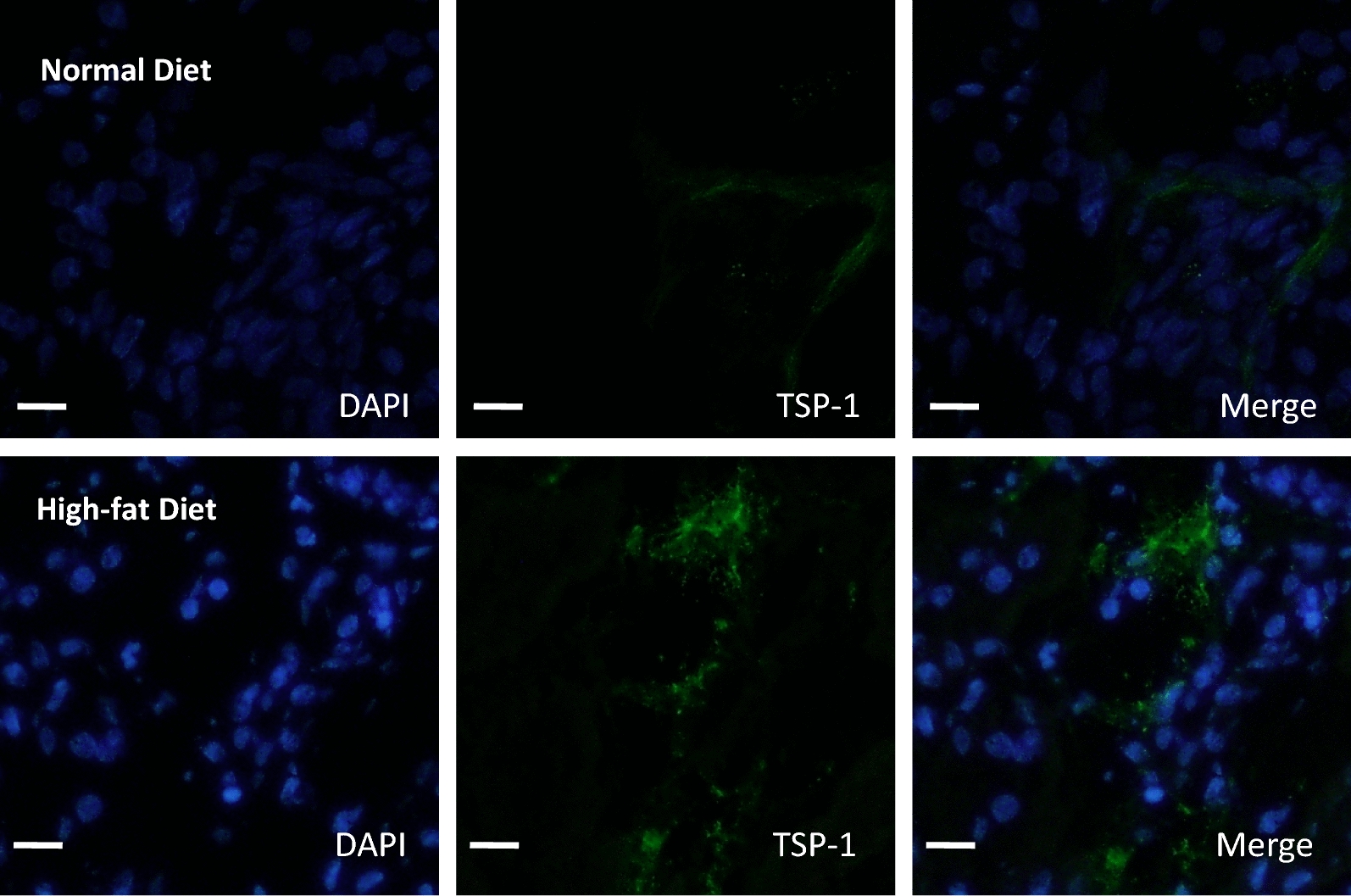
High-fat diet promotes TSP-1 expression in porcine auto-transplanted kidney. TSP-1 expression by immunofluorescence staining was assessed in cortical part from the kidney grafts subjected or not to a hyperlipidic diet. Scale bars represent 100 µm (Magnification ×40; n = 5 in each group)

To investigate the role of TSP-1 in the inhibition of the HIF1a pro-angiogenic pathways induced by hyperlipidic diet, we assessed TSP-1 expression in kidney graft. We observed in the high-fat diet group an increased protein expression of TSP-1 (Fig. 5; Table 2, Additional file 5: Figure S4). These results observed by immunohistological studies showed an elevated staining of TSP-1 in the interstitium, tubules and glomerulus in high-fat diet group but not significant difference in glomerulus parietal cell (Table 2). High-fat diet did not affect VEGF-A and TSP-1 mRNA expressions indicating rather a post-transcriptional regulation of these pathways (Additional file 6: Figure S5).

### High-fat diet induces vascular and tissue remodeling in kidney graft

**Fig. 6:**
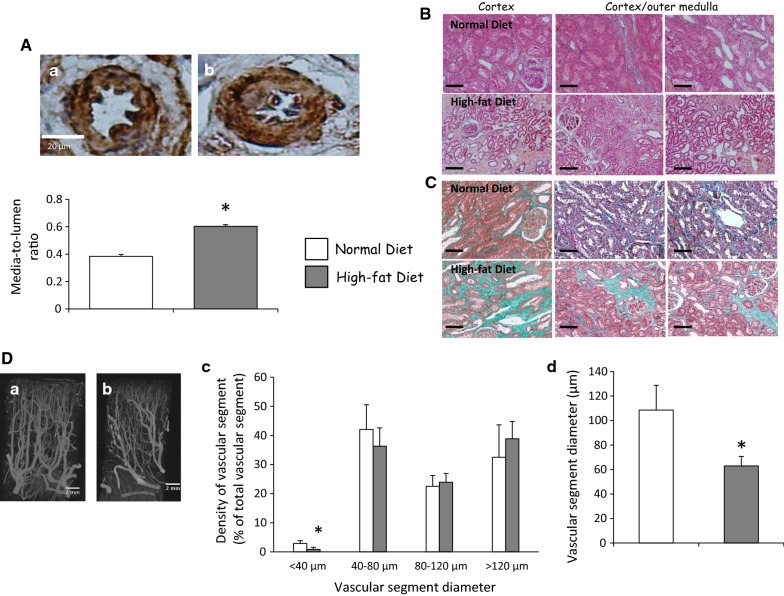
Microvascular remodeling in the kidney graft exposed to high-fat diet. Representative renal α-SMA staining in kidney grafts 3 months after reperfusion in normal (**A**-a) or high-fat diet (**A**-b) groups (up, **A**) and quantification of media-to-lumen ratio (10 vessels by field, down, **A**). We explored in the same groups tubular atrophy by HES staining (Magnif. ×40; **B**); and insterstitial fibrosis by Masson trichrome staining (Magnific. ×40; **C**; n = 5 in each group). Scale bars represent 100 µm. Representative three-dimensional tomographic images of the renal cortical microcirculation in normal diet (**D**-a) or in hyperlipidic diet (**D**-b) and spatial density from different size vascular segments (**D**-c). High-fat diet reduced the average of vascular segment diameter (**D**-d; n = 5 in each group). Values significantly different from the normal diet group are represented by *p < 0.05

**Fig. 7:**
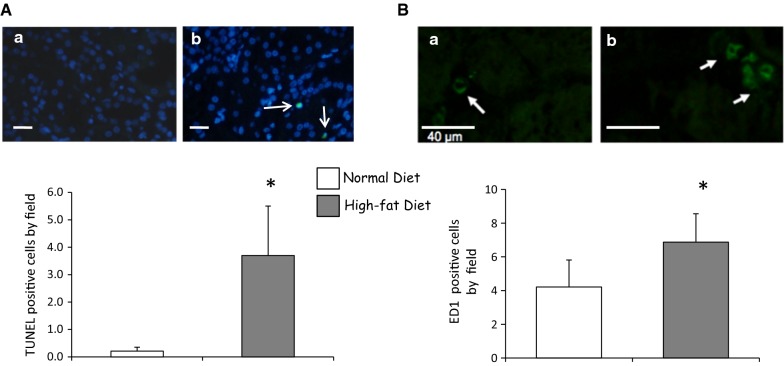
High-fat diet promotes apoptosis and macrophage infiltration in porcine auto-transplanted kidneys. Apoptotic cell death by TUNEL positive cell staining (**A**, scale bars represents 30 µm) and inflammatory ED1 positive cells infiltrated in graft by immunofluorescence staining (**B**, scale bars represents 40 µm) in normal diet (a) or high-fat (b) groups, 3 months after renal auto-transplantation surgery. *p < 0.05 vs. normal diet group; n = 5 in each group

The increase of macrovascular media-to-lumen ratio indicated an intensive vascular remodeling in high-fat diet group compared to normal group (Fig. 6A). In addition, tubular atrophy was increased suggesting deleterious mechanism induced by hyperlipidic diet resulting in non functional fibrous tissue development assessed by Masson trichrome staining (Fig. 6B, C; Table 2) as previously suggested [15]. High resolution micro-computed tomography showed a decrease of the density of vascular segments with a diameter inferior to 40 µm in the hyperlipidic diet kidney grafts, indicating a high microvessel injury (Fig. 6D). These results were supported by aminopeptidase staining indicating a decrease of capillary density in hyperlipidic diet group (Additional file 7; Additional file 8: Figure S6). In the cortex, this experimental group exhibited a reduction of the average vascular segment diameter supporting a vascular remodeling stimulated by high-fat diet (Fig. 6D). These injuries were associated with a pro-inflammatory milieu supported by ED1-positive cells infiltration significantly elevated in dyslipidemic pigs as well as apoptosis stimulation (Fig. 7A, B).

### Hypercholesterolemia the day of surgery affects VEGF-A secretion and promotes chronic graft dysfunction in patients

**Fig. 8:**
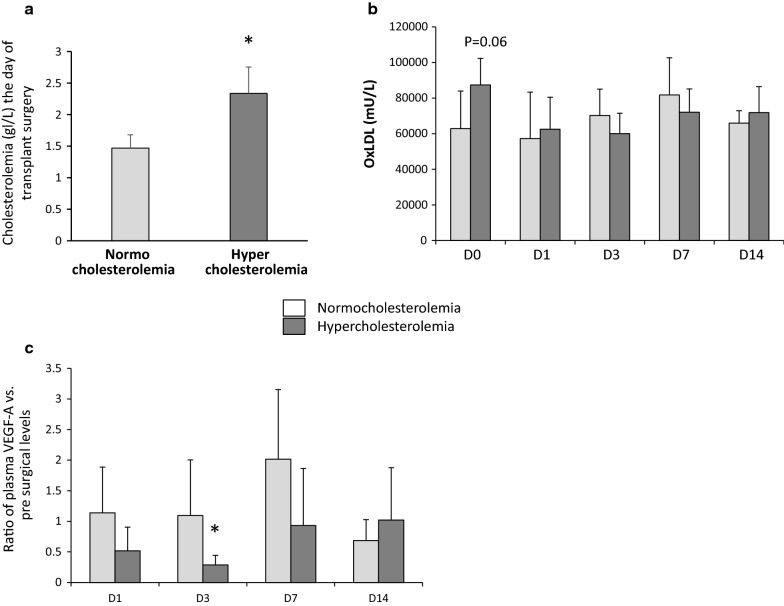
Hypercholesterolemia in renal transplanted patient is associated with an early down regulation of plasmatic VEGF-A. Patients were classified in two groups in relation to their plasma cholesterol levels the day before renal transplantation (**a**; n = 7–9). OxLDL levels were measured in the short term follow up of patients (2 weeks after transplantation) in plasma (**b**; n = 3–9) as well as VEGF-A (**c**; n = 3–8). Values significantly different from the hypocholesterolemic group are represented by *p < 0.05

This single-center study enrolled a total of 16 Caucasian renal transplant recipients. Characteristics of recipients and renal transplant allograft donors are listed in Table 3. All patients received a kidney graft from deceased donors. Using a threshold of 1.80 g/L of plasma cholesterol level, the day before renal transplantation, we classified patients in hyper or normocholesterolemic groups (Fig. 8a). There was no patient death and two grafts loss during the follow up (1 in each group). Plasma OxLDL measurement in the blood samples collected before surgery showed a high level in the hypercholesterolemia group but not reached statistical difference (p = 0.061; Fig. 8b). Interestingly, we observed a decrease of plasmatic VEGF-A level, 1 day after surgery in the hypercholesterolemia group which reached statistical significance at D3 and returned to basal level from D7 (Fig. 8c). The levels of plasma TSP-1 before surgery were increased in the hypercholesterolemia group (Additional file 9: Figure S7). Estimated glomerular filtration rate (eGFR) was similar in both groups up to 3 months post-surgery. However, 1 year after transplantation, the eGFR was significantly reduced in the hypercholesterolemia group (Table 3) and urinary protein excretion increased (Table 3).

**Table 3:** Patient characteristics

	Normocholesterolemia	Hypercholesterolemia
	Donors	Donors
Age (years)	43 ± 9	53 ± 11
Traumatic death (%, n)	29 (2)	33 (3)
Hemorrhagic and ischemic stroke (%, n)	71 (5)	67 (6)
Anastomosis time (min)	42 ± 12	43 ± 9
Cold ischemia time (min)	888 ± 87	870 ± 180
eGFR^a^	81 ± 8	81 ± 6

	Recipients	Recipients
Number	7	9
Age (years)	45 ± 15	50 ± 14
Male gender (%, n)	86 (6)	56 (5)
Dialysis duration (months)	16 ± 9	6 ± 13*
Current smoker (%, n)	71 (5)	22 (2)*
Diabetes mellitus (%, n)	14 (1)	0 (0)
Hypertension (%, n)	86 (6)	78 (7)
Statin (%, n)	57 (4)	44 (4)
Cholesterolemia the day before surgery (mmol/L)	3.80 ± 0.54	6.04 ± 1.08*
Cholesterolemia at 3 months (mmol/L)	4.80 ± 0.47	5.78 ± 1.48
Cholesterolemia at 6 months (mmol/L)	4.32 ± 1.13	4.91 ± 1.19
Cholesterolemia at 12 months (mmol/L)	4.72 ± 1.09	5.10 ± 0.50
eGFR^a^ at 1 month	48 ± 17	45 ± 25
eGFR^a^ at 3 months	53 ± 23	50 ± 18
eGFR^a^ at 12 months	64 ± 13	44 ± 13*
Urinary ratio protein/creatinine (mg/mmol) at 3 month	31.4 ± 21.2	73.9 ± 42.5
Urinary ratio protein/creatinine (mg/mmol) at 12 month	16.7 ± 5.2	78.6 ± 80.4*

Values are mean ± SD

* p < 0.05 vs. normocholesterolemia

^a^eGFR: estimated glomerular filtration rate according to the MDRD formula (mL/min/1.73 m^2^)

## Discussion

Organ shortage in renal transplantation has two major consequences: (1) it pushes transplantation centers to use organs obtained from older donors; (2) it increases the time spent on the waiting list, therefore increasing the average recipient age. This type of donors/recipients is linked with age-related co-morbidity factors such as hypertension or hypercholesterolemia, negatively affecting the transplantation outcome and leading back patients to dialysis [23]. The identification of involved mechanisms could improve the management of recipient in these conditions and promote new therapeutic strategies. Previously, Cui et al. reported for the first time that TSP-1 is upregulated in the kidney from diet-induced obese mice with marked accumulation of TSP-1 in the glomeruli mesangium and tubular system [12]. They also demonstrated that TSP-1 deletion protects mice from obesity-induced renal fibrosis [12]. This detrimental effect was also observed in a syngeneic rat renal transplantation model. Briefly, a CD47 monoclonal antibody, which blocks the interaction between the ligand TSP-1 and its receptor CD47, used during the donor kidney perfusion, induced a marked improvement in post transplant survival [24]. These results were also observed in a renal ischemia reperfusion injury model [25]. In this study, we investigated the early effects of high-fat diet on pro-angiogenic pathway inhibition and TSP-1 expression in a porcine model of renal auto-transplantation with high clinical relevance. This allowed us to focus on I/R injury in the absence of any allogeneic response and easily extrapolate the results to human as porcine and human kidneys have the same multilobular architecture and similar cortical microcirculation [26]. Hypercholesterolemia is associated with increased circulating levels of OxLDL [4]. These oxidized lipoproteins are involved in endothelial cell dysfunction [5], the first cell type subjected to I/R injury in organ transplantation. However, vascular endothelial cells are protected by the release of autocrine signaling molecules such as the pivotal VEGF-A and are well known to proliferate, migrate and regenerate the injured tissue [9]. In a previous study, Sunitinib, a tyrosine kinase inhibitor which inhibits both VEGF and PDGF receptors, prevents chronic rejection changes in experimental kidney transplantation and preserved significantly renal graft function after transplantation highlighting the role of VEGF in early kidney graft function [27].

In the first part of this study, we investigated in endothelial cell model, the effects of OxLDL incubation on TSP-1 expression. We reported, for the first time, that OxLDL promotes TSP-1 secretion in endothelial artery cells according to the increase expression of ADAMTS-1 and decreases the expression of alpha5beta3 integrin known as an integrin required for angiogenesis and endothelial proliferation [28]. TSP-1 uses multiple pathways to modulate endothelial cell migration [29]. TSP-1 is known as a natural antagonist of alpha5beta3 integrin through alpha5beta3 ligation and it has been shown to negatively regulate focal adhesion formation on fibronectin, suggesting that integrin function may be impaired [29]. These results suggest a repressive role of OxLDL production stimulated by hyperlipidic diet on angiogenesis through a TSP-1-dependent pathway underlining by the SiRNA experiment. Renal I/R induces regenerative processes such as angiogenesis which could be affected by OxLDL permanent production. Since our in vitro model relies on the culture on one cell type and is far to mimic the complexity of the kidney physiology, we decided to further test our hypothesis in our porcine model of renal auto-transplantation modulated only by the effect of the diet.

In previous studies, it has been shown that high-fat diet affects renal VEGF-A protein expression and impairs microvascular structures in native porcine kidney without ischemic insults [30, 31]. This aspect is well documented and suggests that high-fat diet in transplant condition could affect pro-angiogenic processes mediated by VEGF-A. We exclusively focused this study on the role of high-fat diet in early vascular regenerative processes after an I/R injury in renal transplantation and excluded the effect of high-fat diet in native kidney since it was previously reported [30, 31]. In pigs fed with a high-fat diet beginning from weaning up, we previously reported an increase in plasma levels of OxLDL at the day of surgery and at 3 months after transplantation [15]. This later increase was concomitant with a rise of total cholesterol levels. The discrepancy between plasma cholesterol and OxLDL levels the day of surgery could be explained by the fast required before surgery and underlined the role of high levels of plasma OxLDL during the early step of reperfusion. During follow up, renal function assessed by creatinine clearance was similar in both groups. This monitoring window for 3 months might be too short to observe significant differences in graft function. However, we observed a significant elevated proteinuria in high-fat diet group at 3 months post-transplantation which is described as a pivotal time point to investigate tissue and vascular remodeling and pro-angiogenic pathways [10, 17].

Renal tissue ischemia is one crucial factor in the development and progression of chronic kidney disease in general. It is known that HIF1a expression is increased in area of severe fibrosis as observed in high-fat diet group indicated by Masson trichrome staining [9]. Decreased oxygen tension activates the stabilization of HIF1a, inducing a large range of pro-survival responses such as VEGF-A expression [32]. However, in this study, high-fat diet promotes a HIF1a stabilization without observed transcriptional effects on VEGF-A expression. In addition, high-fat diet reduced the pro-angiogenic capacity of kidney graft as shown by the repressed expressions of SDF-1 and VEGF-A proteins [9, 28, 33]. Taken together, these results suggest an anti-angiogenic effect of hyperlipidic diet that could be mediated by TSP-1. Neovascularization induced by angiogenic factors is counterbalanced by the effects of angiogenic inhibitors, such as TSP-1, a glycoprotein expressed at very low levels in the healthy renal cortex and upregulated during some renal diseases [12]. Thakar et al. previously reported that TSP-1 null mice exhibited a significant protection against renal ischemia supporting the fact that TSP-1 acts as a regulator of ischemic damages in the kidney [34]. TSP-1 inhibits angiogenesis through its direct effects on endothelial cells and indirect effects on growth-factor mobilization and activation [9]. To investigate the role of TSP-1 in our conditions, we evaluated its expression in grafts. Hyperlipidic diet induced an over-expression of TSP-1 protein, 3 months after transplantation, in glomerulus, intensively marked in tubules and interstitium without mRNA expression change suggesting a post-transcriptional regulation of TSP-1. This hypothesis was supported by ADAMTS-1 overexpression. Indeed, ADAMTS-1 is a soluble matrix metalloprotease molecule that inhibits angiogenesis by mechanisms that may involve direct sequestration of VEGF or release activation of anti-angiogenic thrombospondin-derived peptides such as TSP-1 [35, 36]. ADAMTS-1 was already described to be enhanced in rat proximal tubules after I/R [37] and also in atherosclerosis with macrophage invading the tissue [38]. We observed an increase of ED1-positive macrophages infiltrating interstitium in high-fat diet group, in accordance with the elevated level expression of ADAMTS-1. The significant increase of inflammatory ED-1 positive macrophages in the interstitium of kidney grafts exposed to high OxLDL levels was linked with the profibrotic milieu associated with tubular atrophy. In addition, in this group, TSP-1 could stimulate apoptotic process as suggested by the TUNEL staining increase [39]. In order to investigate the consequences of these pro-inflammatory and regenerative pathways on vascular remodeling, we characterized the cortical microvascularization damages. High-fat diet increased the microvascular rarefaction induced by I/R injury in our renal transplantation model particularly for small vascular segments with diameter inferior to 40 µm according to the decrease of capillary density and the inhibition of pro-angiogenic pathways observed. In addition, the decrease of vascular segment diameter supported a vascular remodeling according to the high ratio of media-to-lumen and fibrosis development.

In order to test the relevance of our results in human transplantation, we investigated the effect of hypercholesterolemia on the systemic levels of the pivotal angiogenic vascular growth factor: VEGF-A in the short-term follow up after renal transplantation. We observed that recipients with high levels of cholesterol the day of surgical procedure, exhibited a decrease of plasmatic levels of VEGF-A during the first 3 days after transplantation suggesting a reduction of the regenerative processes in the graft. In addition, in this preliminary study, we underlined that the level of cholesterolemia the day of surgical procedure could affect graft outcome as indicated by the decrease of filtration function (eGFR) and by the increase of urinary protein excretion in hypercholesterolemic patient, 1 year after reperfusion. Although patient characteristics were similar in both groups, we should notice that other confounding factors could be involved in these differences such as immunosuppressive therapies. Further analyses will be necessary to support the correlation between the increase of TSP-1 levels observed in plasma recipient before surgery and its role on plasma VEGF-A levels early after transplantation.

## Conclusions

Taken together, these results support that: (1) OxLDL are involved in abrogation of regenerative capacity of kidney grafts (2) TSP-1 is overexpressed in porcine kidney grafts exposed to high-fat diet and associated to a repression of pro angiogenic pathways, (3) high-fat diet induces microvascular rarefaction and vascular remodeling and (4) hypercholesterolemic patients have an early decrease of VEGF-A plasma levels after transplantation surgery.

In conclusion, our study underlines the impact of a short term exposition to high-fat diet on the early stage of regenerative processes after renal transplantation, distinct from the chronic injury of atherosclerosis induced by dyslipidemia. In addition, high-fat diet or dyslipidemia in recipient of kidney graft could affect organ outcome identifying TSP-1 as a therapeutic target of interest and emphasizing the need to better control either cholesterol or OxLDL plasma levels in recipient at the early stage of renal transplantation.

## Additional files

**Additional file 1: Table S1.** Primer sequences for RT-qPCR analysis in porcine kidneys.

**Additional file 2: Figure S1.** Expression of TSP1 using western blotting in HAEC (first lane) and in HAEC subjected to Si TSP1 (last lane).

**Additional file 3: Figure S2.** Negative control for immunohistochemistry of HIF1α in renal porcine tissue.

**Additional file 4: Figure S3.** Negative control for immunohistochemistry of VEGF in renal porcine tissue.

**Additional file 5: Figure S4.**  Negative control for immunohistochemistry of TSP1 in renal porcine tissue.

**Additional file 6: Figure S5.** VEGFA, HIF1α, ADAMTS1 and TSP1 mRNA expression by real time quantitative PCR in high-fat or normal diet groups 3 months after auto-transplantation (n=5–6).

**Additional file 7.** Supplementary material for aminopeptidase staining in renal porcine tissue and for plasma TSP1 quantification in human.

**Additional file 8: Figure S6.** Quantification of aminopeptidase by immunostaining from kidneys 3 months after auto-transplantation subjected or not to a hyperlipidic diet.

**Additional file 9: Figure S7.** Hypercholesterolemia is associated with an upregulation of plasmatic TSP-1. Patients were classified in two groups in relation to their plasma cholesterol levels the day before renal transplantation and TSP-1 levels were measured (n=6–7). Values significantly different from the normocholesterolemic group are represented by * p < 0.05.

## Abbreviations

ADAMTS-1: a disintegrin and metalloproteinase with thrombospondin motif-1
αSMA: alpha-smooth muscle actin
eGFR: estimated glomerular filtration rate
HAEC: human aortic endothelial cells
HIF1a: hypoxia-inducible factor 1a
I/R: ischemia reperfusion
LDL: low density lipoprotein
MMP-9: matrix metalloproteinase 9
OxLDL: oxidized LDL
SDF-1: stromal cell-derived factor-1
TGFβ: transforming growth factor β
TSP-1: thrombospondin-1
VEGF-A: vascular endothelial growth factor-A
