# Supplementary material for: Hypercholesterolemia-induced increase in plasma oxidized LDL abrogated pro angiogenic response in kidney grafts
Source: J Transl Med. 2019 Jan 14;17:26. doi: 10.1186/s12967-018-1764-4 (PMC6332834; doi:10.1186/s12967-018-1764-4)
Supplement: Supplementary file 1 — Additional file 1: Table S1. Primer sequences for RT-qPCR analysis in porcine kidneys. [file 12967_2018_1764_MOESM1_ESM.docx]

**Table S1:** Primer sequences for RT-qPCR analysis in porcine kidneys.

| Gene | Forward | Reverse |
| --- | --- | --- |
| ADAMTS-1 | CTTCCTAGCACCCGGTTTCA | AGTAGAAGCAGTGCGCCAAA |
| HIF1a | TGGCAGCAATGACACAGAAAC | GAGGCAGGCAATGGAGACAT |
| VEGF-A | GTGCCCACTGAGGAGTTCAA | AAGGCCCACAGGGATTTTCT |
| TSP-1 | AGCCTCAACAACAGATGCGA | CCCTCACATGGCTTCCCATT |
| RPLPO | AGAAACTGTTGCCTCACATCC | CCTTATTGGCCAGCAGCA |
| SDHA | GAGTTCGTGCAGTTCCACCCTA | CCTCTCACCCTGGCTGTTGATA |
| B-Actin | GATCGTGCGGGACATCAAG | GCCATCTCCTGCTCGAAGTC |
| L19 | AATCGCCAACGCCAACTC | CAGCCCATCTTTGATCAGCTT |
