# Supplementary figures and images for: Hypercholesterolemia-induced increase in plasma oxidized LDL abrogated pro angiogenic response in kidney grafts
Source: J Transl Med. 2019 Jan 14;17:26. doi: 10.1186/s12967-018-1764-4 (PMC6332834; doi:10.1186/s12967-018-1764-4)

## Slide 1
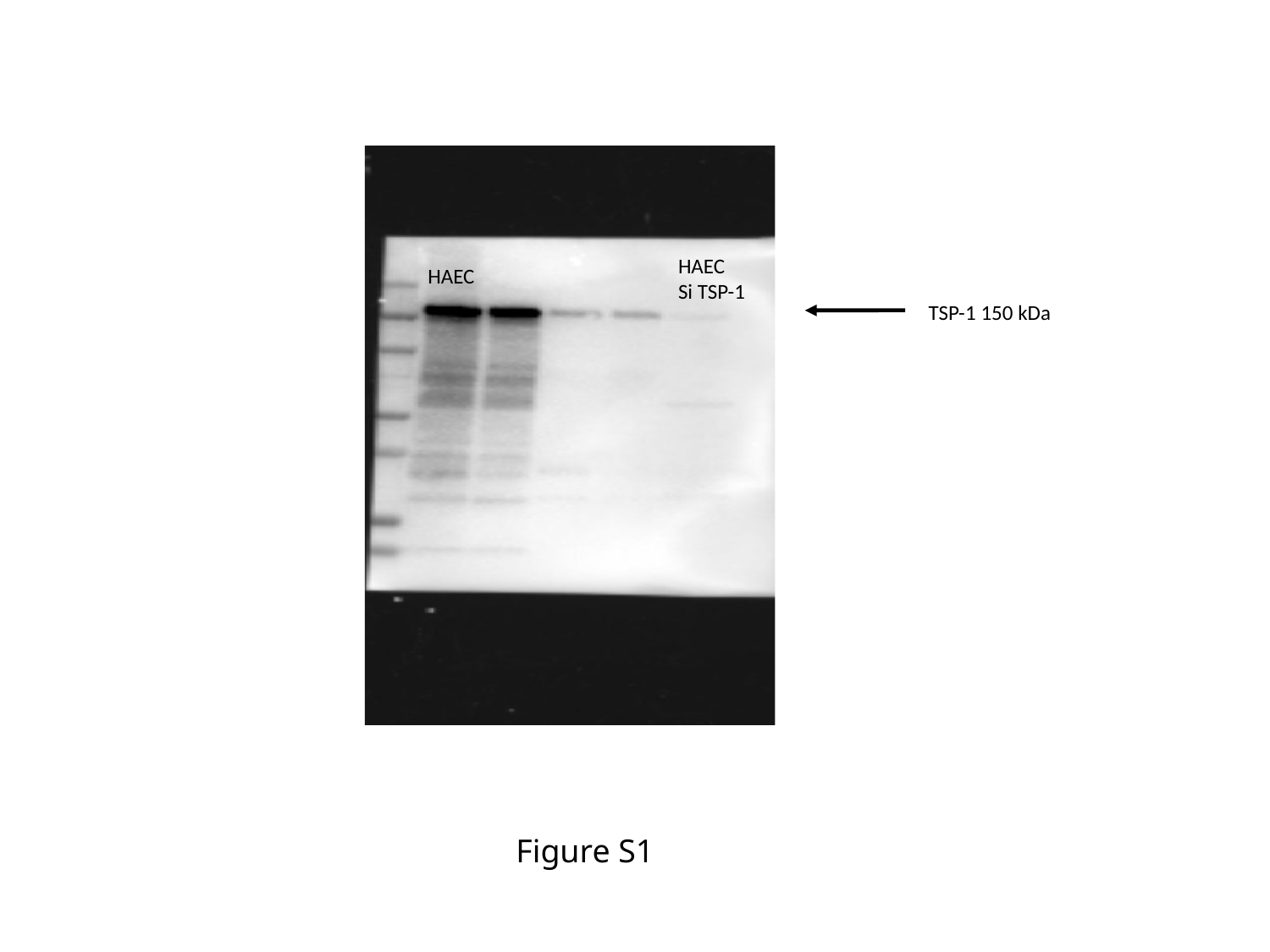

HAEC
Si TSP-1
HAEC
TSP-1 150 kDa
Figure S1

Supplement: Supplementary file 2 — Additional file 2: Figure S1. Expression of TSP1 using western blotting in HAEC (first lane) and in HAEC subjected to Si TSP1 (last lane). [file 12967_2018_1764_MOESM2_ESM.ppt]

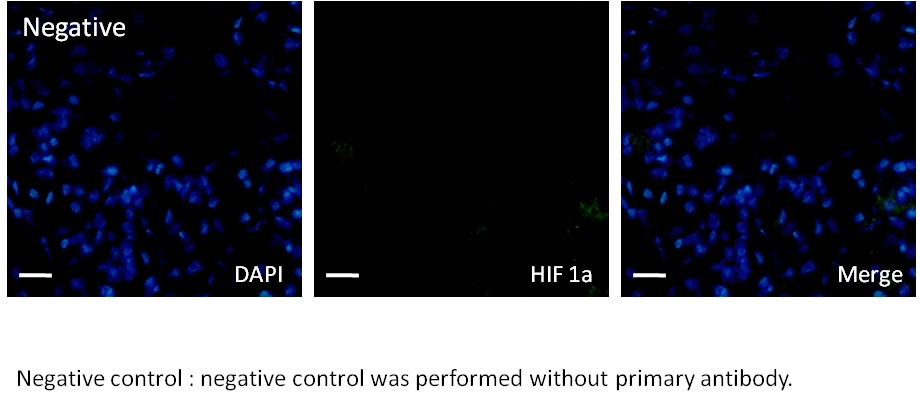

Supplement: Supplementary file 3 — Additional file 3: Figure S2. Negative control for immunohistochemistry of HIF1α in renal porcine tissue. [file 12967_2018_1764_MOESM3_ESM.tif]

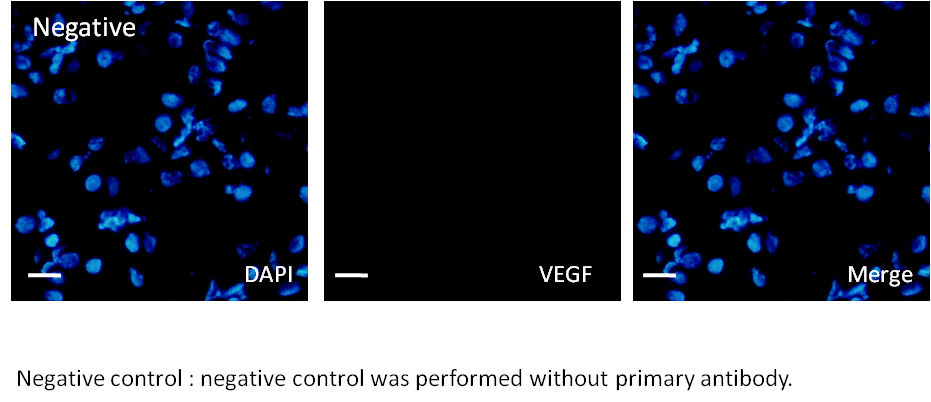

Supplement: Supplementary file 4 — Additional file 4: Figure S3. Negative control for immunohistochemistry of VEGF in renal porcine tissue. [file 12967_2018_1764_MOESM4_ESM.tif]

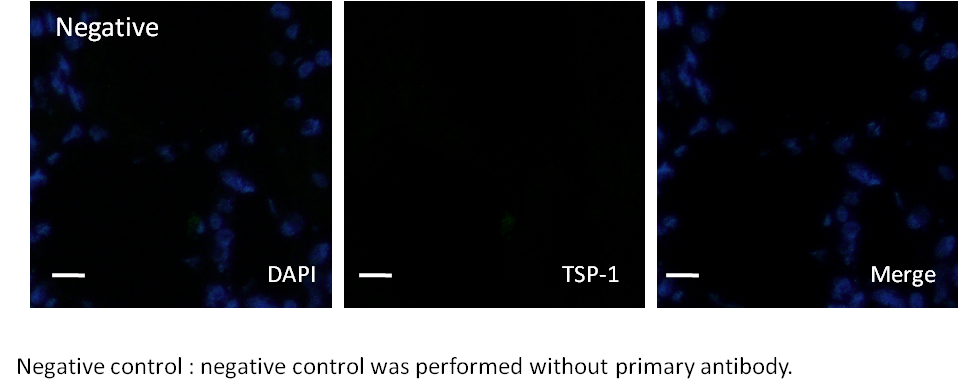

Supplement: Supplementary file 5 — Additional file 5: Figure S4. Negative control for immunohistochemistry of TSP1 in renal porcine tissue. [file 12967_2018_1764_MOESM5_ESM.tif]
