# Supplementary material for: Hypercholesterolemia-induced increase in plasma oxidized LDL abrogated pro angiogenic response in kidney grafts
Source: J Transl Med. 2019 Jan 14;17:26. doi: 10.1186/s12967-018-1764-4 (PMC6332834; doi:10.1186/s12967-018-1764-4)
Supplement: Supplementary file 6 — Additional file 6: Figure S5. VEGFA, HIF1α, ADAMTS1 and TSP1 mRNA expression by real time quantitative PCR in high-fat or normal diet groups 3 months after auto-transplantation (n=5–6). [file 12967_2018_1764_MOESM6_ESM.pptx]

## Slide 1
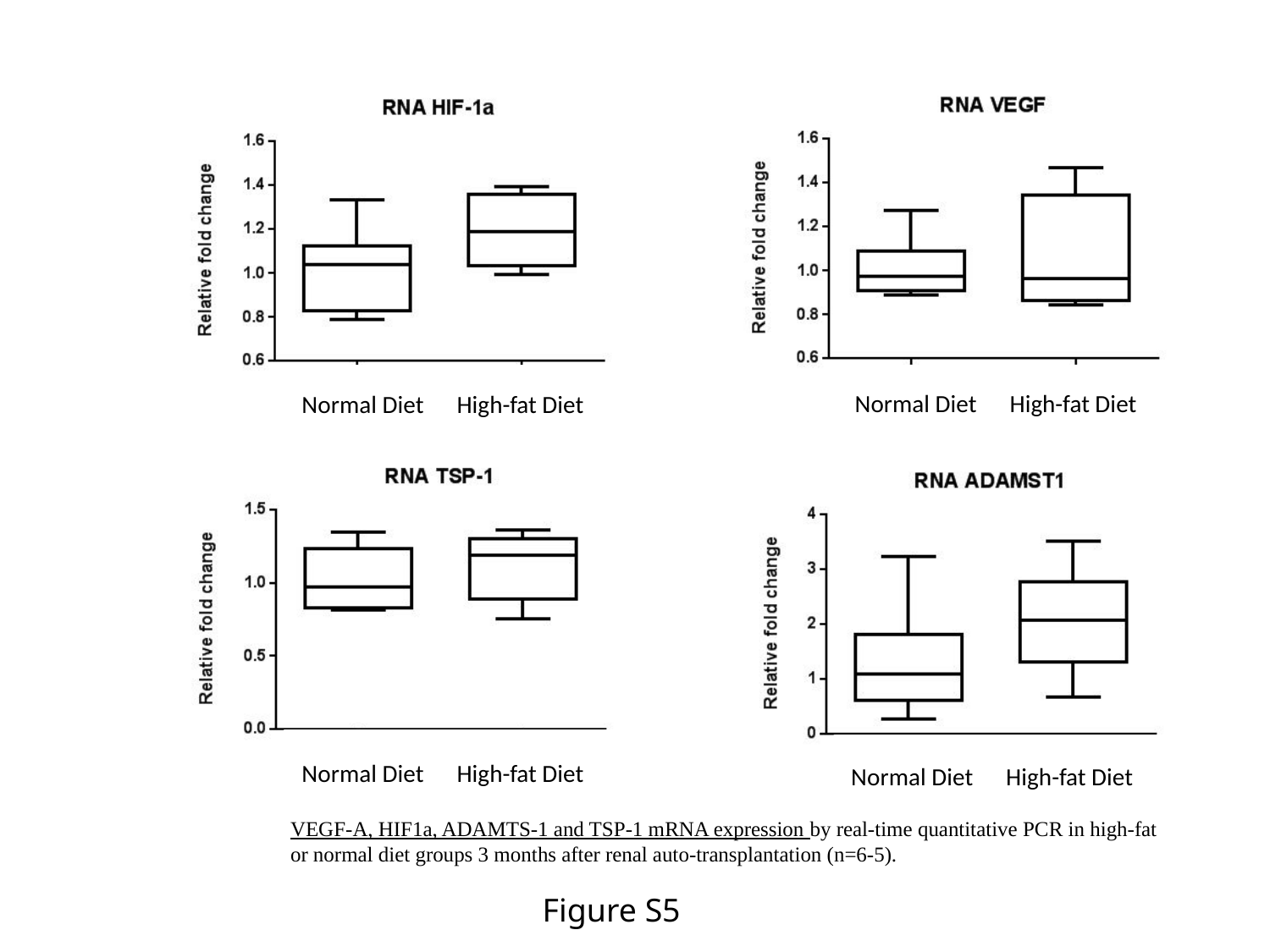

Normal Diet High-fat Diet
Normal Diet High-fat Diet
Normal Diet High-fat Diet
Normal Diet High-fat Diet
VEGF-A, HIF1a, ADAMTS-1 and TSP-1 mRNA expression by real-time quantitative PCR in high-fat or normal diet groups 3 months after renal auto-transplantation (n=6-5).
Figure S5
