# Supplementary material for: Hypercholesterolemia-induced increase in plasma oxidized LDL abrogated pro angiogenic response in kidney grafts
Source: J Transl Med. 2019 Jan 14;17:26. doi: 10.1186/s12967-018-1764-4 (PMC6332834; doi:10.1186/s12967-018-1764-4)
Supplement: Supplementary file 7 — Additional file 7. Supplementary material for aminopeptidase staining in renal porcine tissue and for plasma TSP1 quantification in human. [file 12967_2018_1764_MOESM7_ESM.docx]

**ADDITIONAL MATERIAL**

**Aminopeptidase staining**

Staining evaluation was performed on cortex and outer medulla samples. Paraffin sections were used for aminopeptidase P-positive blood microvessel quantification using an aminopeptidase P antibody (Ref SC-65390, 1/100, Santa Cruz Biotechnology, Santa Cruz, California, USA). This was associated with an appropriate HRP-coupled secondary antibody revealed by diamino benzidine staining.

**Plasma TSP-1 quantification**

Plasma samples were stored at -80°C prior to protein quantification by ELISA for TSP-1 (RD System). The TSP-1 levels were determined in plasma rich in platelets and were expressed with a ratio to total platelet number in blood.
