# Supplementary material for: Hypercholesterolemia-induced increase in plasma oxidized LDL abrogated pro angiogenic response in kidney grafts
Source: J Transl Med. 2019 Jan 14;17:26. doi: 10.1186/s12967-018-1764-4 (PMC6332834; doi:10.1186/s12967-018-1764-4)
Supplement: Supplementary file 8 — Additional file 8: Figure S6. Quantification of aminopeptidase by immunostaining from kidneys 3 months after auto-transplantation subjected or not to a hyperlipidic diet. [file 12967_2018_1764_MOESM8_ESM.pptx]

## Slide 1
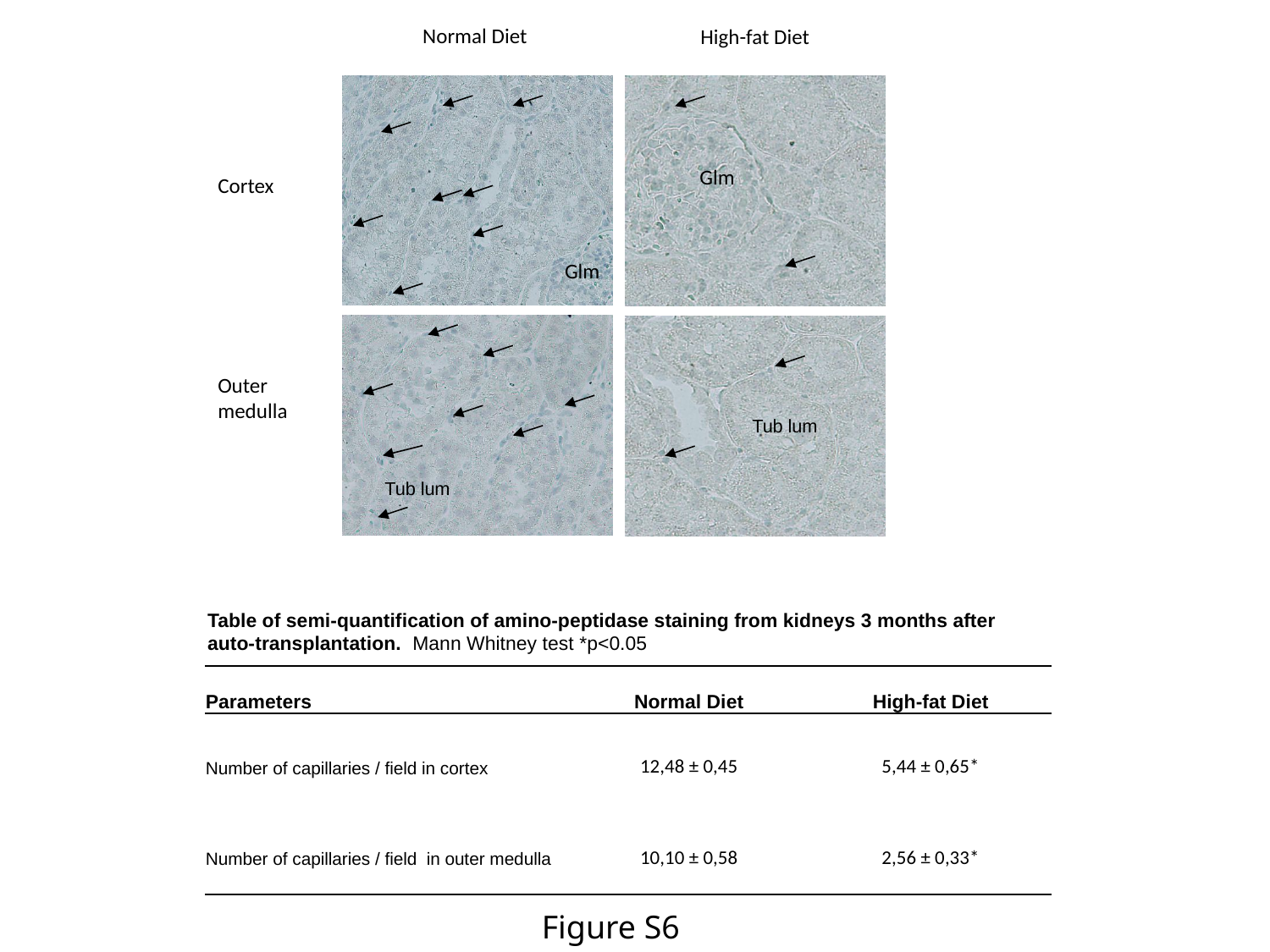

Normal Diet
High-fat Diet
Glm
Cortex
Glm
Outer
medulla
Tub lum
Tub lum
Table of semi-quantification of amino-peptidase staining from kidneys 3 months after auto-transplantation. Mann Whitney test *p<0.05
| Parameters | Normal Diet | High-fat Diet |
| --- | --- | --- |
| Number of capillaries / field in cortex | 12,48 ± 0,45 | 5,44 ± 0,65\* |
| Number of capillaries / field in outer medulla | 10,10 ± 0,58 | 2,56 ± 0,33\* |
Figure S6
