# Supplementary material for: Hypercholesterolemia-induced increase in plasma oxidized LDL abrogated pro angiogenic response in kidney grafts
Source: J Transl Med. 2019 Jan 14;17:26. doi: 10.1186/s12967-018-1764-4 (PMC6332834; doi:10.1186/s12967-018-1764-4)
Supplement: Supplementary file 9 — Additional file 9: Figure S7. Hypercholesterolemia is associated with an upregulation of plasmatic TSP-1. Patients were classified in two groups in relation to their plasma cholesterol levels the day before renal transplantation and TSP-1 levels were measured (n=6–7). Values significantly different from the normocholesterolemic group are represented by * p < 0.05. [file 12967_2018_1764_MOESM9_ESM.pptx]

## Slide 1
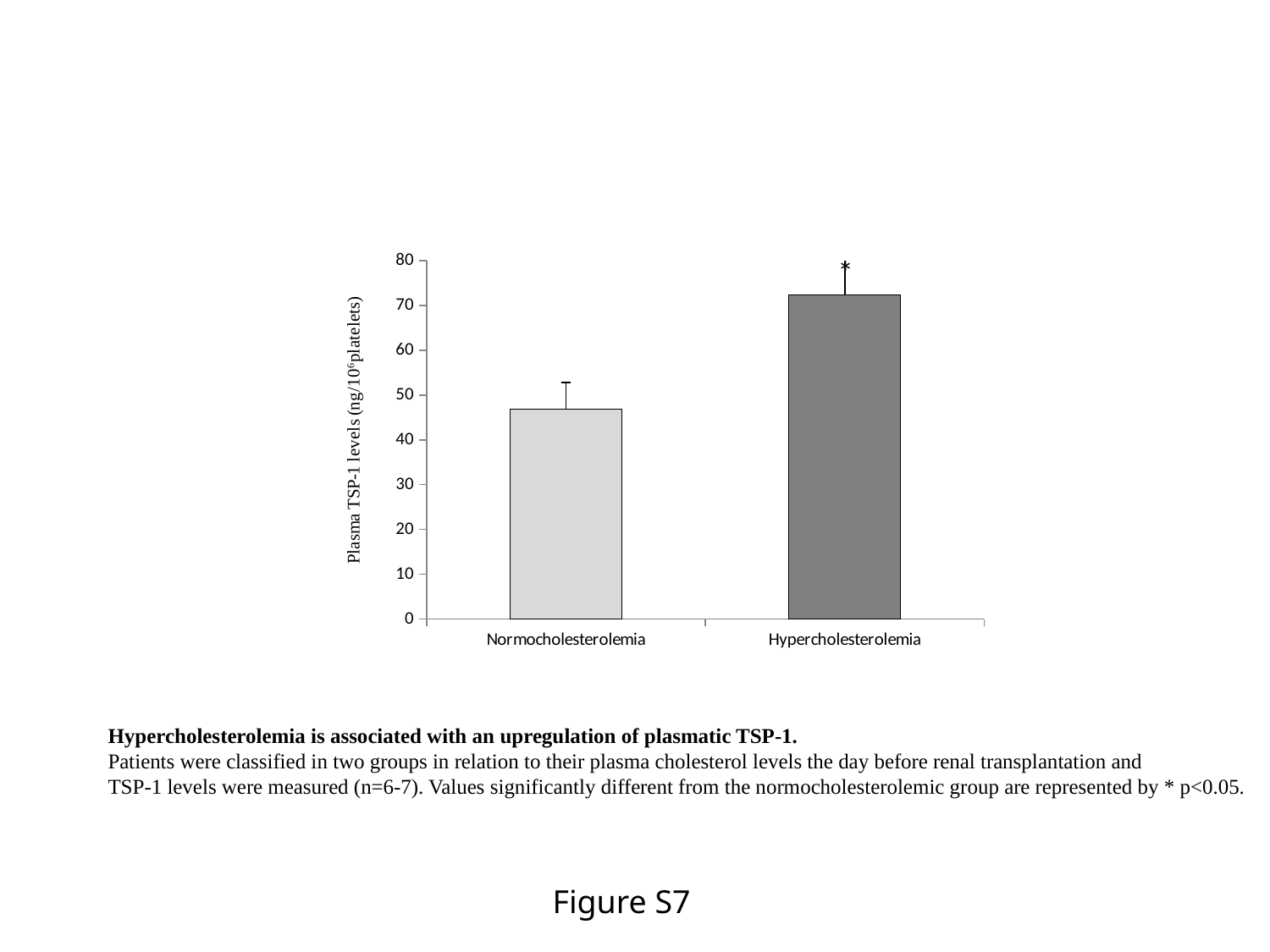

### Chart
| Category | Plasma TSP-1 levels (ng/106platelet) |
|---|---|
| Normocholesterolemia | 46.834503577354845 |
| Hypercholesterolemia | 72.3390039794482 |*
Hypercholesterolemia is associated with an upregulation of plasmatic TSP-1.
Patients were classified in two groups in relation to their plasma cholesterol levels the day before renal transplantation and
TSP-1 levels were measured (n=6-7). Values significantly different from the normocholesterolemic group are represented by * p<0.05.
Figure S7
